# Application of Preoperative Ultrasonography in the Percutaneous Minimally Invasive Repair of Acute Closed Achilles Tendon Rupture

**DOI:** 10.1155/2023/8956803

**Published:** 2023-01-09

**Authors:** Zhuang Wang, Weiwei Chen, Honglei Jia, Fangning Hu, Bomin Wang, Yongliang Yang, Fanxiao Liu

**Affiliations:** ^1^Department of Orthopaedics, Shandong Provincial Hospital Affiliated to Shandong First Medical University, Jinan, Shandong Province, China; ^2^Department of Radiology, Jinan Central Hospital Affiliated to Shandong First Medical University, Jinan, Shandong Province, China

## Abstract

Percutaneous minimally invasive surgery involving Achilles tendon (AT) repair has the advantages of a low rerupture rate and fewer postoperative complications. However, due to the inability to operate under direct vision, the injury of the small saphenous vein (SSV) and sural nerve (SN) remains largely a high risk involving many challenges. We propose to introduce the preoperative application and advantages of ultrasonography in percutaneous minimally invasive surgery for acute AT rupture. Our results indicated that ultrasonography could locate the position of the SN more accurately and reduce the risk of iatrogenic nerve injury. Compared with the traditional surface markers, the preoperative localization and marking of AT, SSV, and SN in ultrasonography significantly reduced the risk of intraoperative accidental injury to blood vessels and nerves, which could reduce postoperative complications and promote early rehabilitation of patients. We ultimately exploit the properties of ultrasonography in percutaneous minimally invasive surgery to treat Achilles tendon rupture.

## 1. Introduction

The Achilles tendon (AT) is the strongest and largest tendon and the most frequently ruptured tendon in the human body [1]. Achilles tendon repair accounts for approximately 40% of all tendon repair operations [2]. The treatment strategies for acute AT rupture include conservative and surgical treatment, and the latter includes open surgery and minimally invasive surgery with their advantages and disadvantages [3–5]. Up to now, the ideal method is still controversial. Recent studies [6–9] indicated that surgical treatment of acute AT rupture is becoming more and more popular, especially in athletes with high demands on the function of the ankle. Open surgery could make the stump of the broken tendon repair and anastomosis accurate, which allows the patient functional exercise early with a low risk of rerupture [10]. Although the risk of vascular and nerve injury around AT is relatively low, open surgery is associated with numerous postoperative complications, including infection, skin necrosis, delayed healing, or scar adhesion, which may be related to excessive intraoperative skin and soft tissue. Cetti et al. reported that open surgery has the risk of skin adhesion, infection, and nerve injury, and the incidence of complications is as high as 34.1% [11].

To avoid incision-related complications, minimally invasive repair of AT rupture has become a hot topic in clinical research. Our previous study revealed a satisfactory result in the treatment of acute AT rupture using minimally invasive repair [12]. A meta-analysis confirmed that there was no significant difference between minimally invasive repair and open repair in terms of complications such as relapse, tissue adhesion, deep infection, sural nerve (SN) injury, and deep venous thrombosis (DVT), but the subjective excellent result of minimally invasive surgery was 3 times higher than that of open surgery, and the superficial infection rate decreased significantly [4]. It has also been suggested that percutaneous repair is a feasible alternative to open surgery due to the high complication rate and high cost of treatment [13]. Percutaneous repair of AT rupture can significantly reduce the surgical trauma of the affected foot, improve the prognosis, and decrease the overall complications of the operation with a short hospital stay and low cost. However, the disadvantages cannot be ignored. Because the passaging of AT and SN is anatomically close together, and percutaneous minimally invasive repair cannot be performed under direct vision, the greatest risk of percutaneous repair is SN injury.

Body surface markers are often used to locate the SN before minimally invasive Achilles tendon surgery [14], but they cannot accurately indicate the course of SN (the line between the midpoint of the popliteal fossa and the midpoint of the lateral ankle to the Achilles tendon). Therefore, the injury to the SN cannot be completely avoided. A physical study of the anatomical relationship between the SN and the edge of the AT revealed that the intersection of the SN and the AT was 6.55-16 cm proximal to the AT attachment point, implying various courses of SN among different individuals [15]. One meta-analysis involving 9 studies demonstrated that the incidence of SN injury in the percutaneous minimally invasive treatment group was 5.5%, while that in the open surgery group was only 1.2% [7]. Klein et al. reported that the incidence of SN injury caused by minimally invasive percutaneous repair was close to 13% [16]. In view of the above defects, numerous researchers have proposed the use of ultrasonography to determine the anatomical location of SN, small saphenous vein (SSV), and AT [17, 18]. Giannetti et al. conducted minimally invasive percutaneous AT surgery using ultrasonography intraoperatively, and no complications of SN injury occurred during 13 months of follow-up [19]. Two previous studies performed by our team confirmed that ultrasound is highly effective and accurate in detecting soft tissue injuries such as full-thickness rotator cuff tears [20, 21]. In view of the above reasons, ultrasound is used for preoperative localization of the anatomical structure of the ruptured AT in this study.

Anatomical studies have shown that SSV always passes medially along with the SN [22]. Therefore, in this study, the location of the SSV was used to assess the approximate location of the SN, and the differences were compared between preoperative ultrasound localization-assisted minimally invasive repair of acute Achilles tendon rupture and traditional preoperative surface marker localization as well as the advantages of preoperative ultrasound and the effect of short-term postoperative recovery.

## 2. Materials and Methods

### 2.1. Patients' Selection Criteria

Inclusion criteria are as follows: (1) patients with acute closed AT rupture; (2) AT rupture within 3 weeks; (3) there is an obvious gap at the ruptured ends; Thompson test (+); (4) the insertion distance of AT should be longer than 2 cm to the broken end; (5) sports injury; no history of chronic AT pain or Achilles tendinitis.

Exclusion criteria are as follows: (1) patients with open AT rupture; (2) rupture of AT for more than 3 weeks or rerupture of AT; (3) history of steroid use (partial closure or long-term oral administration); (4) other injuries around the AT, such as ankle fracture; (5) history of chronic AT pain or Achilles tendinitis.

According to the inclusion criteria and exclusion criteria, the patients with acute closed AT rupture from March 2020 to December 2020 in Shandong Provincial Hospital Affiliated to Shandong First Medical University were included in the study.

### 2.2. Preoperative Anatomical Structure Marking and Ultrasonic Localization

**Figure 1:**
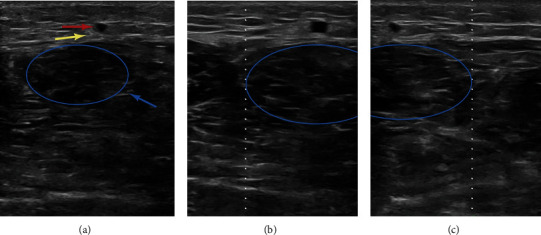
A male patient, 28 years old, with an Achilles tendon rupture in a football match. The pictures show the anatomical structure on the plane of the proximal end of the tendon (9 cm from the proximal end of the broken end of the Achilles tendon) examined by ultrasound. In this plane, the small saphenous vein and sural nerve are located directly above the Achilles tendon. (a) The blue arrow shows tendons; the red arrow shows the small saphenous vein; the yellow arrow shows the sural nerve. (b) The dotted line shows the lateral edge of the Achilles tendon. (c) The dotted line shows the medial edge of the Achilles tendon.

**Figure 2:**
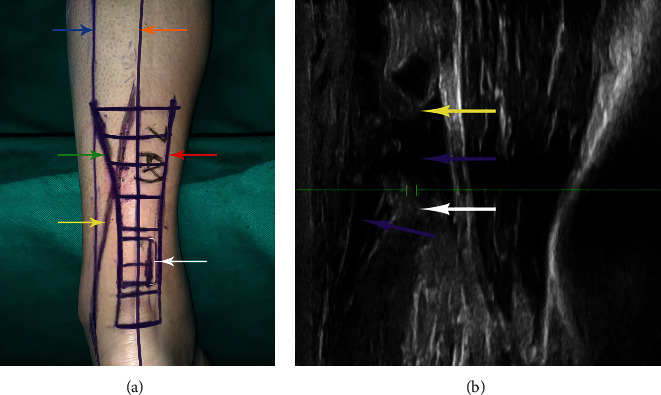
(a) The anatomical structure was located preoperatively by body surface localization and ultrasonography. The blue arrow shows the course of the small saphenous vein and sural nerve drawn through the body surface markers. The yellow arrow shows the exact course of small saphenous vein and sural nerve located by ultrasonography. The orange arrow represents the midline of the Achilles tendon measured by ultrasonography. The red arrow shows the medial edge of the Achilles tendon on ultrasonography. The green arrow shows the lateral edge of the Achilles tendon on ultrasonography. The white arrow shows the broken end of the Achilles tendon on ultrasonography. (b) Ultrasonography image of ruptured Achilles tendon in sagittal view. The yellow arrow shows proximal Achilles tendon stump. The white arrow shows distal Achilles tendon stump. The purple arrow shows hematoma.

All patients received epidural anesthesia. The tourniquet is not necessary because of less bleeding in the minimally invasive surgery. All patients were operated by the same surgical team who had not received any special training from radiologists. After successful anesthesia, the patient was placed in the prone position, and a point was drawn every 2 cm from the insertion point of the AT to the proximal end of the affected limb to the muscle belly, and a horizontal line was drawn through each point. First, we marked the course of SN and SSV according to traditional body surface markers (the line between the midpoint of the popliteal fossa and the lateral ankle to the AT). Then, the ultrasonic machine is used with a high-frequency linear array probe (L15-4B) and a frequency of 4-15 MHz (Wisonic Navis, Shenzhen, China). The distal and proximal stumps of AT were first probed to understand the specific location and damage condition of the stumps and marked, and then, the specific positions of the medial and lateral border and the midpoint of AT, SSV, and SN were marked on the body surface in each plane from proximal to distal (Figures 1(a)–1(c)). By connecting all points on the plane, the accurate position of AT and the midline of AT, SSV, and SN can be obtained (Figure 2). To make the picture more intuitive, we used image processing software to process the picture (Adobe Photoshop 2022, Adobe Systems, USA). It can be seen that the closer to the proximal end of the limb, the greater the error between body surface localization (clinical position) and ultrasound localization (actual position) of AT, SSV, and SN. Taking the midline of AT as the “0” point, the distance from the clinical position and actual position of SSV and SN to the midpoint of AT as well as the distance between the lateral and medial sides was measured on each plane. All related data were recorded in Table 1. Since SN along the SSV, ultrasound is superior to neuroimaging for vascular imaging; only the distance from the SSV to the midpoint of the AT needs to be recorded.

### 2.3. Surgical Methods

After data recording, surgery was performed according to the anatomical locations marked by ultrasound. In this study, a minimally invasive Bunnell suture was used. A vertical skin incision of approximately 2 cm in length is made along the rupture of AT, and the skin and subcutaneous layers are incised layer and layer. If the paratenon is still intact, identify and cut it. The distal and proximal stumps of AT were exposed, and the distal and proximal stumps of AT were pulled out of the incision with arterial forceps, and the hematoma and necrotic tissue of the stump of AT and the surrounding tissue was removed with tissue scissors. Longitudinal puncture incisions were selected on the medial and lateral sides of AT at 1 cm, 3 cm, and 5 cm proximal to the incision. Since SN is accompanied by the lateral side of SSV, it should be noted that the incision on the lateral of AT should be inside the SSV measured by ultrasound to injury to SSV and SN. From proximal end to distal end, under the guidance of a suture grasper closure device (Beijing HangTian KaDi Technology R&D Institute, Beijing, China), No. 2 Ethibond suture (polyester unabsorbable suture, Johnson & Johnson, USA) was threaded from the lateral AT longitudinal incision through the center of AT transversely through the medial longitudinal incision of AT. The end of the suture is then guided again by the suture grasper closure device and passed diagonally through the distal tendon incision. The sutures at both ends were repeated under the guidance of the trocar, and the oblique cross suture was performed on three horizontal planes at the proximal end of AT and finally passed through the longitudinal incision at the rupture of AT for use. A longitudinal puncture incision was made on the inside and outside of AT at 1 cm and 3 cm perpendicular to the skin incision at the distal stump of AT, and another No. 2 Ethibond suture was sutured in the same way. Finally, the suture was also passed through the longitudinal incision where AT was ruptured. At the incision in the ankle and knee flexion, proximal and distal sutures are pulled tight and tied tightly on either side of the torn tendon. No emptiness felt on the stump of AT, Thompson test (-). After repeated flushing with normal saline, the stump of AT and collateral ligament was sutured with 2-0 absorbable suture (Johnson & Johnson, USA), and the incision was sutured with 3-0 absorbable suture (Johnson & Johnson, USA).

### 2.4. Postoperative Treatment and Rehabilitation

After the operation, the ankle joints of patients were immobilized with a short-leg cast of 20°-30° plantar flexion for 3 weeks. During this period, exercises of the hips, knees, and toes are allowed, such as active contraction and relaxation of the quadriceps femoris and triceps crus. After 5-6 weeks, the short-leg cast was removed, and the flexio-adjustable walking boots were replaced. The patients continue to wear it at the same flexion angle for 4 weeks. During this time, the patients were told to walk with crutches. After the foot reaches neutral flexion, allow the patients to gradually exercise the heel to improve strength and ankle range of motion and allow to active movement in regular shoes.

### 2.5. Postoperative Follow-Up

Ultrasound follow-up was performed on the patients at 3, 6, and 12 weeks after operation to guide postoperative rehabilitation training. Follow-up indicators mainly include as follows: sural nerve palsy, rerupture, deep infection, superficial infection, large hematoma, and DVT. Patients were followed up every 3 months postoperatively. Patients were assessed preoperatively and at the last follow-up visit using the American Orthopaedic Foot and Ankle Society (AOFAS) scale [23].

### 2.6. Statistical Analysis

SPSS 25.0 was used for statistical analysis. The measurement data conforming to the normal distribution were expressed as mean ± standard deviation, and the paired sample *t*-test was used for two groups of data in the same plane. A *p* value <0.05 indicated a statistically significant difference.

## 3. Results

A total of 16 patients with acute closed AT rupture were included, involving 13 males and 3 females, aged 22-39 years old. The left AT ruptured in 6 cases, and the right was in 10 cases. All were sports injuries involving basketball, football, badminton, and volleyball. The main clinical symptoms were inability to walk and plantar flexion. Ultrasound or magnetic resonance imaging (MRI) was performed on admission to determine the complete rupture and location of AT rupture. The distance from the broken end to the calcaneal insertion point was 2-6 cm, with an average of 2.8 cm. The time from injury to operation was 1-5 days, with an average of 3.6 days. The mean follow-up time was 19.56 ± 3.84 months (15-24 months). The mean tear location at the calcaneal insertion was 3.8 ± 1.62 cm (range: 2.2-5.6 cm). The basic information of the 16 included patients is shown in Table 2.

### 3.1. Measurement Result

The actual position of SN at 10 cm from the insertion of the calcaneus was located on the medial side of the lateral border of AT (0.94 ± 0.19 cm < 1.73 ± 0.19 cm, *p* < 0.001), which was quite different from the position of the Achilles tendon located by the body surface marker (2.84 ± 0.37 cm > 1.73 ± 0.19 cm, *p* < 0.001). The distance between the two gradually decreases as you get closer to the distal end of AT. At 8 cm from the calcaneal insertion, the actual position of SN became the lateral border of AT (2.11 ± 0.34 cm > 1.46 ± 0.16 cm, *p* < 0.001), but the distance from the clinial position to actual position was too far away (3.11 ± 0.35 cm, *p* < 0.001). The same result was obtained for calcaneal insertion of 6 cm (Table 3). The distance from SN to the midpoint of AT and 1/2 of the width of AT measured by the two methods was assessed by paired sample *t*-test. The test results support our above conclusion (*p* < 0.001). Another paired sample *t*-test showed that there were significant differences between the actual and clinical positions of SN in different planes (*p* < 0.001) (Table 4).

### 3.2. Clinical Function and Complication

During the follow-up period, no SN injury such as foot numbness and paresthesia was found in all patients. The preoperative AOFAS score was 59.17 ± 5.31 cm (range: 52-72), which increased to 98.92 ± 1.63 cm (range: 95-100) at the last follow-up. The difference was statistically significant. At the last follow-up, all patients walked normally, with good heel elevation and the same activity intensity as before AT rupture. Reexaminated ultrasound showed good continuity of AT.

## 4. Discussion

This study involving 16 patients evaluated the location of SN and SSV at three levels using body surface markers and ultrasonography, which revealed that compared with ultrasonography, the application of body surface markers to determine the location of SN and SSV has a great difference. Preoperative ultrasonography localization can accurately determine the location of SN and SSV, which greatly reduces the risk of injury in surgery. In addition, ultrasonography is simple with a low cost. It has great application and promotion value in the percutaneous minimally invasive repair of acute closed AT rupture.

Although the percutaneous minimally invasive surgery could greatly reduce the complications caused by open surgery incision, the inability to perform surgery under direct vision increases the probability of SN injury [13, 24, 25]. At present, minimally invasive surgery mostly locates the course of SN through body surface markers before surgery. However, the actual course of SN cannot be determined through body surface marker, and the probability of accidental SN injury remains high. Klein et al. reported 13% of SN injuries using the Ma-Griffith technique to repair freshly ruptured AT [16]. These studies showed that there is a large difference in the localization of SN only using body surface markers in different patients. Iatrogenic SN injury negatively affects the popularity of percutaneous minimally invasive surgery and the quality of life of postoperative patients.

To avoid SN injury, Flavin et al. [25] suggested ultrasonography as a method to determine the localization of SN. Ultrasonography has a good effect on soft tissue imaging. Compared to MRI, ultrasound is relatively inexpensive and does not involve radiation. It can be performed at the bedside or in the operating room. The operation is simple and flexible. A study conducted by Wang et al. [9] undergoing ultrasound-guided repair of AT rupture showed that ultrasonography had the advantages of no radiation, no tissue injury, and good soft tissue visualization. Moreover, minimally invasive treatment of AT rupture could reduce complications compared with open surgery. Ultrasound not only has a good resolution for tendon and other soft tissues but also can clearly display the shape and continuity of tendons and can also display the height, degree, and severity of the broken end, which has a good auxiliary role in the diagnosis and treatment of acute AT rupture [26].

The SN is the main cutaneous nerve of the lower extremity. It passes through the posterolateral side of the calf, attaches to SSV, and innervates the lateral border of the hindfoot, the lateral border of the midfoot, and the ankle joint. The sural intestine is usually composed of four named parts: the medial sural cutaneous nerve, the lateral sural cutaneous nerve, the fibular branch of communication, and the sural nerve [27]. Kammar et al. [17] reported that using ultrasound, the average distance between the SN and the lateral of AT at the insertion of AT and the proximal 4, 8, and 11 cm was 21.48 mm, 11.47 mm, 5.8 mm, and 0.81 mm, respectively, and there were variations in different individuals. In patients with shorter leg and older age, the SN was closer to AT. This increases the intraoperative risk of iatrogenic injury. In the above situation, if only simple body surface markers are used to determine the approximate position of SN, it is very easy to cause injury. Although there are variations in the course of the SN in individual patients, the anatomy of the sural nerve of 24 Egyptian legs and feet by Eid and Hegazy [22] found that all SSVs passed medially along AN. Ultrasound images blood vessels better than nerves; therefore, much more attention should be paid when selecting an insertion point at the lateral border of AT. As long as the specific location of SSVs can be determined, puncture and suture at the proximal stump of SSV can well avoid damage to the injury of SN. In the present study, our findings demonstrated that all SSVs passed medial to SN, and all SNs run along and close SSV, which is consistent with that of Eid and Hegazy's study [22]. For the above reasons, we only need to record the distance between clinical location and ultrasound location of SSV to the midpoint of AT, so as to compare the accuracy of SSV and SN positioning between the two positioning methods. In addition, preoperative ultrasonography of SN in 16 patients revealed that the proximal-to-distal path of SN obliquely traverses the surface of AT from medial to lateral. Therefore, the closer to the proximal end of the affected limb, the more consideration should be given to whether SN will be injured or compressed when selecting the puncture site. Our study also confirmed that the closer to the proximal extremity, the greater the difference between the body surface location (clinical position) and the ultrasound location (actual position) of AT, SSV, and SN. Due to the different width of AT in different patients, the midline of AT is also different. In the paired sample *t*-test, in order to correct for the effect of the width of AT, this study used the ratio of the distance from SN surface anchor point to the midpoint of AT to the width of AT and the distance from ultrasound anchor point to the midpoint of AT to the width of AT statistical analysis (Table 4), which fully indicates that there is a large difference in the localization of sural nerve based solely on body surface markers (*p* < 0.001), and the ultrasonic localization of SN has guided significance for assisted percutaneous minimally invasive AT repair. This also reminds us that the proximal outer edge of the ruptured Achilles tendon is most likely to injure SN. The closer it is to the calcaneus, the closer SN is laterally, and the farther it is from AT. After the ultrasound machine is removed before surgery, the body surface position of sural nerve and the actual walking path under ultrasonography are recorded and compared. The puncture point can be selected on the inner side of actual walking path of SN, and the puncture and suture can be achieved safely and accurately, and good surgical results can be achieved without excessive use of ultrasonography. Percutaneous minimally invasive AT repair has many advantages over open surgery, but previous literature reports show that its biggest disadvantage is the high probability of SN injury [3, 4, 28, 29]. Preoperative ultrasound was used to locate SN, and a minimally invasive Bunnell suture was used to repair AT rupture, which solved the problem of SN injury well. Ultrasonography has the advantages of simple operation, accurate positioning, safety and noninvasiveness, short time-consuming, and high promotion value, which has guiding effect on the next step rehabilitation treatment of patients. According to the above methods, all 16 patients underwent percutaneous minimally invasive AT repair, which achieved ideal results. All patients had no symptoms of sural nerve injury during follow-up.

However, this study also has some shortcomings. First of all, it should be noted that this was a retrospective, self-controlled study with a relatively small number of patients; the next step is to conduct the study in a larger sample to obtain more convincing results. Second, the length of the calf of the research object was not included in the research index. The effect of calf length on ultrasound localization of sural nerve cannot be demonstrated. Finally, the surgical methods used in this study were all minimally invasive Bunnell suture, which failed to demonstrate the safety and effectiveness of this surgical method.

## 5. Conclusion

For the Achilles tendon minimally invasive surgery, there is a large error in locating sural nerve and small saphenous vein using body surface markers. Preoperative ultrasonography localization can accurately determine the location of sural nerve and small saphenous vein, which greatly reduces the risk of injury in surgery. In addition, ultrasonography, as a simple and low-cost method, has great application and popularization value in percutaneous minimally invasive repair of acute closed Achilles tendon rupture.

## Figures and Tables

**Table 1:** The basic information of the included patients.

Variable	Study subjects (*n* = 16)
Age (years, mean ± SD, range)	31.0 ± 6.51 (23-43)
Male/female (%)	(81.25%)/(18.75%)
Injured limb (left or right, %)	
Left	6
Right	10
Cause of injury (*n*, %)	
Basketball	4
Football	3
Badminton	7
Volleyball	2
Tear site (distance from the calcaneal insertion) (cm, mean ± SD)	3.8 ± 1.62
Time from injury to operation (days, mean ± SD)	3.6 ± 1.3

**Table 2:** Data of clinical and actual small saphenous vein positions and ratios to the width of Achilles tendon.

Patients	Checkpoint 1 (cm)		Checkpoint 2 (cm)		Checkpoint 3 (cm)	
	CP/WAT	AP/WAT	CP/WAT	AP/WAT	CP/WAT	AP/WAT
1	2.85/3.50	1.05/3.50	3.20/3.00	1.90/3.00	3.35/2.50	2.35/2.50
2	2.70/3.40	1.10/3.40	2.90/2.80	1.90/2.80	3.00/2.40	2.20/2.40
3	3.20/3.80	0.90/3.80	3.35/3.10	2.25/3.10	3.60/2.60	2.80/2.60
4	2.40/3.00	1.00/3.00	2.65/2.50	1.65/2.50	2.85/2.10	2.05/2.10
5	3.10/3.80	1.20/3.80	3.05/2.90	2.05/2.90	3.30/2.60	2.70/2.60
6	2.65/3.30	0.95/3.30	2.95/2.70	1.85/2.70	2.70/2.20	2.00/2.20
7	3.25/3.90	0.75/3.90	3.55/3.30	2.45/3.30	3.80/2.80	3.00/2.80
8	2.95/3.50	0.95/3.50	3.25/2.90	2.15/2.90	3.40/2.40	2.50/2.40
9	2.20/2.80	1.00/2.80	2.55/2.30	1.65/2.30	2.45/1.90	1.65/1.90
10	3.40/4.00	0.50/4.00	3.80/3.40	2.70/3.40	4.05/2.90	3.15/2.90
11	2.90/3.40	1.10/3.40	3.15/3.10	2.35/3.10	3.35/2.50	2.45/2.50
12	2.70/3.20	0.80/3.20	3.25/2.90	2.25/2.90	3.25/2.30	2.15/2.30
13	2.35/2.90	0.95/2.90	2.90/2.80	1.90/2.80	2.80/2.00	1.90/2.00
14	2.50/3.20	1.00/3.20	2.70/2.60	1.70/2.60	3.00/2.20	2.20/2.20
15	3.00/3.60	1.20/3.60	2.95/2.90	2.35/2.90	3.60/2.80	2.90/2.80
16	3.35/4.10	0.65/4.10	3.55/3.50	2.65/3.50	3.75/3.10	3.15/3.10

Checkpoint 1; 10 cm proximal to calcaneal insertion; checkpoint 2: 8 cm proximal to calcaneal insertion; checkpoint 3: 6 cm proximal to calcaneal insertion. Take the midline of Achilles tendon as the “0” point. CP: clinical position: the distance from the sural nerve located by body surface location point to the midpoint of Achilles tendon; AP: actual position: the distance from the sural nerve located by ultrasonography to the midpoint of Achilles tendon; WAT; width of Achilles tendon: the distance from the medial edge to the lateral edge of the Achilles tendon on each plane.

**Table 3:** The actual and clinical positions of the sural nerves and their relationship with the midpoint of the Achilles tendon.

	Checkpoint 1			Checkpoint 2			Checkpoint 3		
	CP	AP	Half of WAT	CP	AP	Half of WAT	CP	AP	Half of WAT
Mean	2.84	0.94	1.73	3.11	2.11	1.46	3.27	2.45	1.23
SD	0.37	0.19	0.19	0.35	0.34	0.159	0.44	0.46	0.17

Checkpoint 1: 10 cm proximal to calcaneal insertion; checkpoint 2: 8 cm proximal to calcaneal insertion; checkpoint 3: 6 cm proximal to calcaneal insertion. Take the midline of Achilles tendon as the “0” point. CP: clinical position: the distance from the sural nerve located by body surface location point to the midpoint of Achilles tendon; AP: actual position: the distance from the sural nerve located by ultrasonography to the midpoint of Achilles tendon; WAT: width of Achilles tendon: the distance from the medial edge to the lateral edge of the Achilles tendon on each plane.

**Table 4:** Paired sample *t*-test.

Groups		Mean	SD	*p* value
Checkpoint 1	Clinical position/width of Achilles tendon-actual position/width of Achilles tendon	0.54195	0.08251	< 0.001
Checkpoint 2	Clinical position/width of Achilles tendon-actual position/width of Achilles tendon	0.34586	0.06052	<0.001
Checkpoint 3	Clinical position/width of Achilles tendon-actual position/width of Achilles tendon	0.34116	0.07836	<0.001

Checkpoint 1: 10 cm proximal to calcaneal insertion; checkpoint 2: 8 cm proximal to calcaneal insertion; checkpoint 3: 6 cm proximal to calcaneal insertion. SD: standard deviation.

## Data Availability

The datasets used and/or analyzed during the current study are available from the corresponding authors on reasonable request.
